# Supplementary material for: Long-Term Pulmonary Dysfunction by Hyperoxia Exposure during Severe Viral Lower Respiratory Tract Infection in Mice
Source: Pathogens. 2022 Nov 12;11(11):1334. doi: 10.3390/pathogens11111334 (PMC9696792; doi:10.3390/pathogens11111334)
Supplement: Supplementary file 1 [file pathogens-11-01334-s001.zip › pathogens-2012924-supplementary.pdf]

Supplementary Materials:

INDEX

**Content**	**Page**
Table S1: Amplification of the *Sh*-gene 7 days after intranasal inoculation with the pneumonia virus of mice	2
Figure S1: Semi-survival of mice up to post-natal day 28 when end-point outcomes were assessed for the different experimental conditions	3
Figure S2: Inspiratory capacity for the different experimental conditions	4
Figure S3: Pressure-driven pressure volume loops for the different experimental conditions	5
Figure S4: Static compliance (Cst), Salazar-Knowles parameter K (K) and area of pressure volume loops (Hysteresis) derived from the pressure-driven pressure volume loops for the different experimental conditions	6

Table S1. Amplification of the *Sh*-gene 7 days after intranasal inoculation with the pneumonia virus of mice

	PVM 1:500 (N=10)	PVM 1:1000 (N=8)	PVM 1:2000 (N=10)	RPMI (N=5)
ΔCt[a], median [IQR]	-10.3 [-14.5 – -6.1]	-10.4 [-15.3 – -3.7]	-10.6 [-13.1 – -4.0]	N/A[b]

Table S1. Amplification of the pneumonia virus of mice Sh-gene 7 days after intranasal inoculation with varying doses (dilution of PVM stock in RPMI is shown). a, ΔCt is calculated based on the *POLR2A* housekeeping-gene; b, sh-gene specific amplification could not be detected in RPMI controls. PVM, pneumonia virus of mice; N, number of mice; IQR, interquartile range.

Figure S1. Semi-survival of mice up to post-natal day 28 when end-point outcomes were assessed for the different experimental conditions

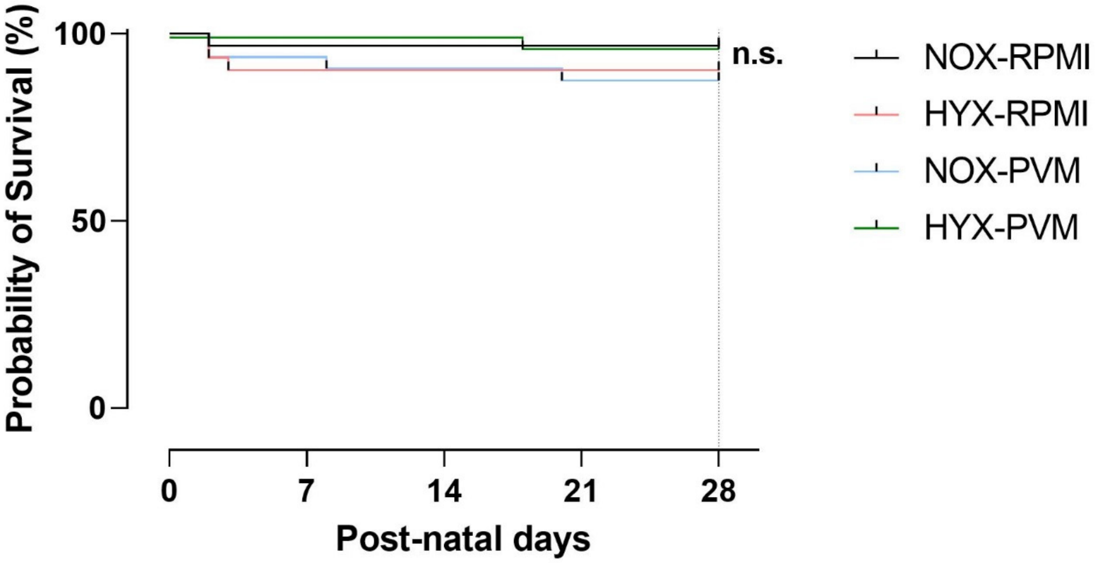

Semi-survival of mice up to post-natal day 28 when end-point outcomes were assessed for the different experimental conditions: RPMI non-infected controls and PVM-infected mice with either normoxia exposure (NOX: 21% O_2) or hyperoxia exposure (HYX: 85% O_2). Difference in survival was assessed by log-rank test. NOX-RPMI (N=30), HYX-RPMI (N=28), NOX-PVM (N=28), HYX-PVM (N=31). Survival curves indicate mice that were found dead in the cage, or mice that were cannibalized by mothers. Under animal ethics guidelines, mice would have been euthanized when scored for distress or lack of condition. However, no mice were scored as being in distress or in poor condition before being found dead.

Figure S2. Inspiratory capacity for the different experimental conditions

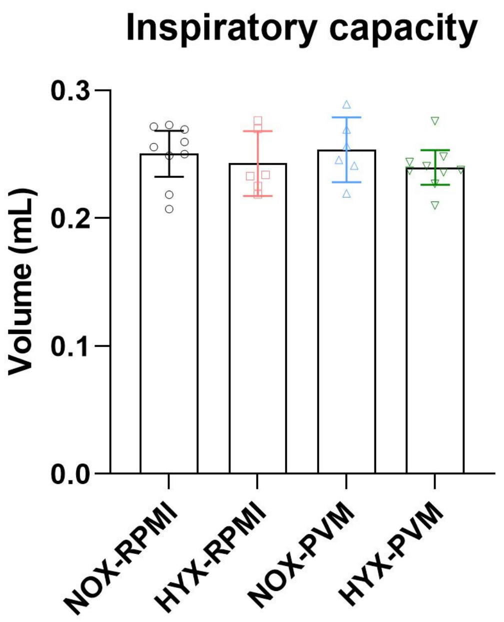

Inspiratory capacity for the different experimental conditions: RPMI non-infected controls and PVM-infected mice with either normoxia exposure (NOX: 21% O_2) or hyperoxia exposure (HYX: 85% O_2). Mean with corresponding 95% confidence interval are shown. NOX-RPMI (N=9), HYX-RPMI (N=6), NOX-PVM (N=6), HYX-PVM (N=9).

Figure S3. Pressure-driven pressure volume loops for the different experimental conditions

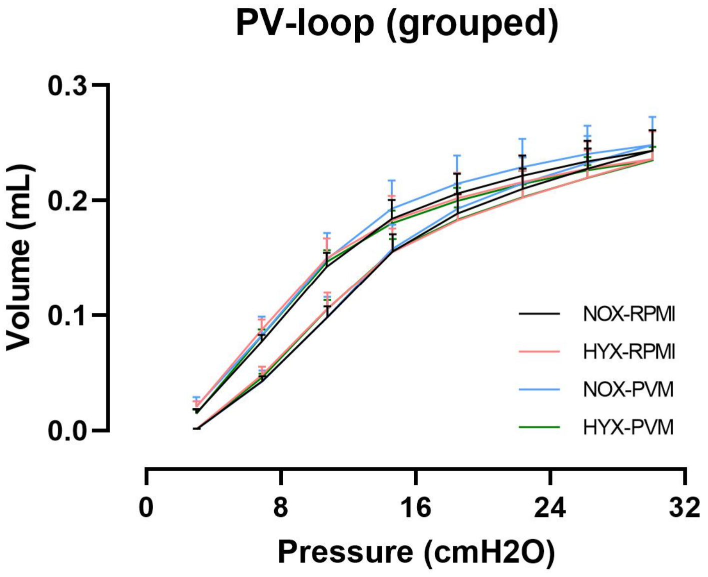

Pressure-driven pressure volume loops for the different experimental conditions: RPMI non-infected controls and PVM-infected mice with either normoxia exposure (NOX: 21% O_2) or hyperoxia exposure (HYX: 85% O_2). Mean with corresponding 95% confidence interval are shown. NOX-RPMI (N=9), HYX-RPMI (N=6), NOX-PVM (N=6), HYX-PVM (N=9).

Figure S4. Static compliance (Cst), Salazar-Knowles parameter K (K) and area of pressure volume loops (Hysteresis) derived from the pressure-driven pressure volume loops for the different experimental conditions

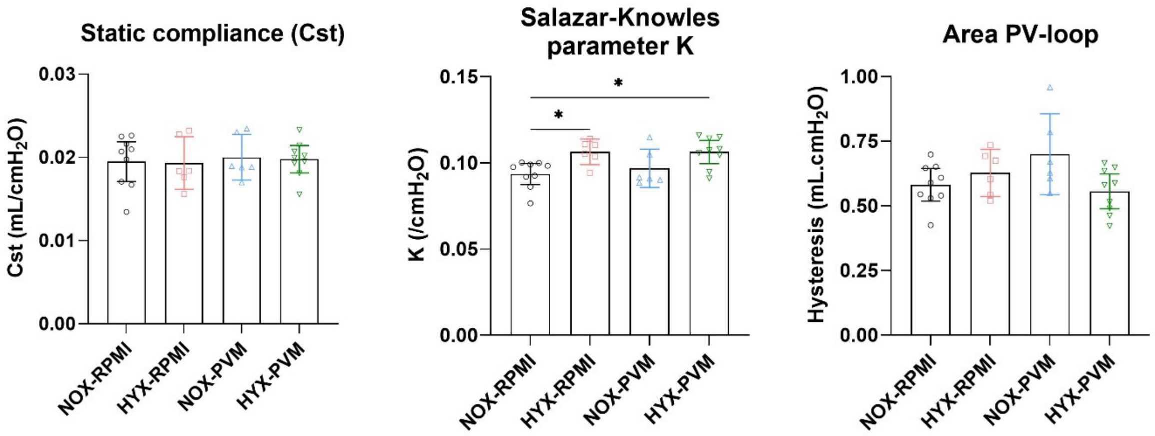

Static compliance (Cst), Salazar-Knowles parameter K (K) and area of pressure volume loops (Hysteresis) derived from the pressure-driven pressure volume loops for the different experimental conditions: RPMI non-infected controls and PVM-infected mice with either normoxia exposure (NOX: 21% O_2) or hyperoxia exposure (HYX: 85% O_2). Mean with corresponding 95% confidence interval are shown. NOX-RPMI (N=9), HYX-RPMI (N=6), NOX-PVM (N=6), HYX-PVM (N=9). *, $P < .05$.
